# Crocodylians evolved scattered multi-sensory micro-organs

**DOI:** 10.1186/2041-9139-4-19

**Published:** 2013-07-02

**Authors:** Nicolas Di-Poï, Michel C Milinkovitch

**Affiliations:** 1 Department of Genetics & Evolution, Laboratory of Artificial & Natural Evolution (LANE), University of Geneva, 1211, Geneva 4, Switzerland

**Keywords:** Crocodylians, Integument, Scale, Sensory organ, ISO, DPR

## Abstract

**Background:**

During their evolution towards a complete life cycle on land, stem reptiles developed both an impermeable multi-layered keratinized epidermis and skin appendages (scales) providing mechanical, thermal, and chemical protection. Previous studies have demonstrated that, despite the presence of a particularly armored skin, crocodylians have exquisite mechanosensory abilities thanks to the presence of small integumentary sensory organs (ISOs) distributed on postcranial and/or cranial scales.

**Results:**

Here, we analyze and compare the structure, innervation, embryonic morphogenesis and sensory functions of postcranial, cranial, and lingual sensory organs of the Nile crocodile (*Crocodylus niloticus*) and the spectacled caiman (*Caiman crocodilus*). Our molecular analyses indicate that sensory neurons of crocodylian ISOs express a large repertoire of transduction channels involved in mechano-, thermo-, and chemosensory functions, and our electrophysiological analyses confirm that each ISO exhibits a combined sensitivity to mechanical, thermal and pH stimuli (but not hyper-osmotic salinity), making them remarkable multi-sensorial micro-organs with no equivalent in the sensory systems of other vertebrate lineages. We also show that ISOs all exhibit similar morphologies and modes of development, despite forming at different stages of scale morphogenesis across the body.

**Conclusions:**

The ancestral vertebrate diffused sensory system of the skin was transformed in the crocodylian lineages into an array of discrete multi-sensory micro-organs innervated by multiple pools of sensory neurons. This discretization of skin sensory expression sites is unique among vertebrates and allowed crocodylians to develop a highly-armored, but very sensitive, skin.

## Background

Vertebrates exhibit diverse skin appendages, such as hair follicles, mammary glands, feathers, and scales that constitute class-defining features within amniotes (mammals, birds, and reptiles). Stem reptiles were the first vertebrates to evolve a complete life cycle on land thanks to the development of both the amniotic egg and a complex multi-layered keratinized epidermis. The latter provides an efficient barrier against water loss and ultraviolet irradiation. Various scale types (keratinized and/or ossified scales) and arrangements have further diversified for better mechanical, thermal, and chemical protection [1-3]. In this respect, crocodylians that include the true crocodiles (*Crocodylidae*), the gharials (*Gavialidae*), and the alligators and caimans (*Alligatoridae*), are remarkable as they have large and particularly robust epidermal scales. The toughness of crocodylian skin and scales results from both the strongly collagenous dermis and the presence of a hard β-keratinized, stratified epidermis [4-8]. In addition, crocodylian skin is yet harder and thicker (i) in dorso-caudal regions where it contains osteoderms (bony plates) [9,10] and (ii) on the face and jaws, where the scales are not genetically controlled developmental units, but emerge from a process entirely analogous to physical cracking of a living tissue in a stress field [11].

The ability of living organisms to sense environmental cues is critical to survival. All eukaryotes, including animals, plants, and fungi, have developed specialized receptors responding, among others, to light (photoreceptors), mechanical parameters (mechanoreceptors), chemical stimuli (odor and taste receptors), relative and absolute changes in temperature (thermoreceptors), and damage (nociceptors, that is, those responsible for pain perception). Sensory neurons are typically coupled to activation of ion channels, thereby transducing specific stimuli into electrical signals. These ion channels are activated either directly by the stimulus, such as observed for many mechanoreceptors, or indirectly by second messengers of a transduction cascade, such as observed for odorant and light stimuli [12,13].

All orders of amniotes have developed multiple specialized cutaneous sensory receptors (also called papillae, corpuscles, or pits) that vary in localization within the skin (that is, in the epidermis, the dermis, and/or the oral mucosa) and distribution across the body [14]. Whereas histological studies have revealed the diverse morphological organizations of these structures (Merckel cells, Meissner-like corpuscles, lamellated receptors, touch papillae, taste buds, and free nerve-endings), only a few studies have focused on their functional physiology [15-21]. Among reptiles, crocodylians are well-provided with sensory organs. Besides good vision and hearing, they exhibit small melanin-pigmented integumentary organs distributed on virtually all scales (cranial and postcranial) in gharials and crocodylids, while they are absent from postcranial scales in alligators and caimans [22-24]. These organs were initially named integumentary sensory organs (ISOs) [25], but were renamed dome pressure receptors (DPRs) when behavioral analyses demonstrated their mechanosensory abilities [26]. These results were confirmed and extended with anatomical and electrophysiological analyses [27], indicating that cranial and body ISOs in juvenile crocodylians constitute a mechanosensory system with high resolution (given the large number and wide distribution of ISOs on the skin) and high sensitivity (possibly greater than that observed in primate fingertips). Behavioral experiments were inconclusive to identify whether these organs may assist in osmoregulation [28-30], in addition to lingual salt-secreting glands described in the oral cavity of some crocodylian species [31-35]. Although electrophysiology analyses [27] indicate that postcranial ISOs do not respond to changes in osmolarity, the sensitivity of the ISOs to other stimuli has not been investigated. Finally, morphological and molecular developmental analyses of ISOs in crocodylian embryos, and a direct comparison of their structure and innervation pattern in different crocodylian species and body regions are lacking.

Here, we analyze and compare the structure, innervation, embryonic morphogenesis, and sensory function of postcranial, cranial and lingual sensory organs in two crocodylian species differing by their distribution of ISOs (that is, cranial and postcranial, or cranial only): the Nile crocodile (*Crocodylus niloticus (C. niloticus)*) and the spectacled caiman (*Caiman crocodilus*). Our work demonstrates that crocodylians evolved a highly armored, but very sensitive skin through the assembly of multiple sensory receptors (diffusely distributed in other amniotes) into discrete and scattered multi-function micro-organs.

## Methods

Maintenance of, and experiments on crocodylians were approved by the Geneva Canton ethical regulation authority (authorization 1008/3693/1) and performed according to Swiss law.

### Crocodylian embryos

Fertilized eggs of *C. niloticus* and *Caiman crocodilus* were incubated on a moistened vermiculite substrate at 29.5°C. Crocodylian embryos were removed at different embryonic days post-oviposition and were staged based on their external morphology according to Ferguson [36,37]. Correspondences between incubation time (E) and Ferguson stages (FS) were as follows: E34 = FS 19, E38 = FS 20, E45 = FS 22, E55 = FS 23, and E70 = FS 25.

### Whole mount *in situ* hybridization

Crocodylian embryos at different developmental stages were fixed overnight in 4% paraformaldehyde (PFA) in PBS at 4°C, dehydrated though a series of methanol/PBS solutions (25%, 50%, 75% and 100% methanol), and stored at −20°C until hybridization. Whole mount *in situ* hybridization (WMISH) was performed as in [38] at a temperature of 62°C and with species-specific digoxigenin-labelled antisense riboprobes corresponding to Nile crocodile *Asic1* (1,001 bp, CDS region), *Asic2* (716 bp, CDS region), *Trek1* (784 bp, CDS region), *Trpc1* (1,080 bp, CDS region), *Trpa1* (565 bp, CDS region), *Trpm8* (397 bp, CDS region), *Trpv1* (638 bp, CDS region), *Trpv2* (1018 bp, CDS region), *Trpv3* (709 bp, CDS region), or *Trpv4* (665 bp, CDS region) genes. Corresponding sense riboprobes were used as negative controls. After WMISH, embryos were fixed in 4% PFA, cryoprotected in 30% sucrose, embedded in optimum cutting temperature (OCT) compound and cryosectioned at 20 μm.

### Histology and immunofluorescence

Embryonic skin tissues from different body locations were fixed overnight at 4°C with 4% PFA before alcohol dehydration and paraffin embedding. Tissues were then sectioned at 8 μm and stained with H&E according to standard protocols. Immunofluorescence staining on skin paraffin sections was carried out as follows: enzyme- (Protease XXV for 30 minutes at room temperature) or heat-induced (0.01 M citrate buffer, pH 6.0, for 20 minutes at 95°C) epitope retrieval; blockage for 60 minutes with 5% normal goat serum; incubation overnight at 4°C with one of the following primary antibodies known to recognize reptile and/or chicken epitopes: anti-pan-cadherin (1:500, Abcam, Cambridge, UK), anti-pan-α-cytokeratin (1:50, Thermo Scientific), anti-acetylated tubulin (1:200, Sigma), anti-PGP9.5 (1:20, Abcam), anti-SP (1:50, GenWay, San Diego, CA, USA), anti-NSE (1:100, GenWay), anti-GAP43 (1:400, Abnova, Taipei City, Taiwan), anti-NEUN (1:50, GeneTex, Irvine, CA, USA), anti-NEFH (1:100, Thermo Scientific, Waltham, MA, USA), anti-SST (1:50; Abcam), or anti-NPY (1:100; Abcam); and finally, incubation with the Alexa Fluor-conjugated secondary antibody (Alexa Fluor-488 or −568, Invitrogen, Carlsbad, CA, USA) for one hour. Samples were mounted with Vectashield mounting medium (Vector Laboratories, Burlingame, CA, USA) containing 4′,6′-diamidino-2-phenylindole (DAPI). The functionality and specificity of all primary antibodies was further confirmed by performing positive controls on crocodylian embryonic brain tissues. For double immunofluorescent staining, the two primary antibodies coming from different animal species were mixed together and two secondary antibodies were used.

### Semiquantitative reverse transcription-PCR

Total RNA from neonatal Nile crocodile and spectacled caiman tissues (total brain, jaw skin, dorsal skin, ventral skin, neck skin, and tongue) was isolated using the RNeasy mini kit (Qiagen, Venlo, Netherlands), according to the manufacturer’s instructions. cDNA was generated by reverse transcription (RT) using 2.5 μM of oligo(dT) primer and 1 μg of total RNA (SuperScript kit, Invitrogen), and analyzed by semiquantitative PCR using the FastStart PCR system (Roche, Basel, Switzerland). PCR primers, identical for the two crocodylian species, and used to amplify short fragments of 200 to 600 bp, were designed on the basis of species-specific gene sequences obtained either from http://www.reptilian-transcriptomes.org/[39] or from sequencing PCR fragments (amplified with various combinations of degenerated primers corresponding to sequences that are highly conserved in vertebrates). Gene orthology was predicted based on multi-species sequence alignments and high conservation with genome sequences from closely related amniotes, including birds (chicken, zebra finch and turkey; >88% sequence identity) and other crocodylians (American alligator, saltwater crocodile and Indian gharial [40]; >96% sequence identity). The following primers were used: *Asic1* forward primer (Fw), 5′- CCA GAC MTT YGT GTC CTG CCA −3′; *Asic1* reverse primer (Rv), 5′- GAK GCY TTG CTG GGG ATC TT −3′; *Asic2* Fw, 5′- CCC CAG CAA GAC TTC AGC CAA G −3′; *Asic2* Rv, 5′- CAC CAA GTA AGG CTG CCA CTT C −3′; *Trek1* Fw, 5′- GTA GTG GCA GCA ATA AAT GCA G −3′; *Trek1* Rv, 5′- CTG ATC CAC CTG CAA CAT AG −3′; *Trpc1* Fw, 5′- CTG TCA TTT TAG CTG CTC ATC G −3′; *Trpc1* Rv, 5′- TAG AGC TGG TGG TAT ACA TG −3′; *Trpa1* Fw, 5′- TCA TTT RCA GTG GGA ATG TGG −3′; *Trpa1* Rv, 5′- CAT RAA TCC ACA GTA TCT TGG −3′; *Trpm8* Fw, 5′- ACC GAC ATT GGG AGT CTG CTG A −3′; *Trpm8* Rv, 5′- TGC AAA GGG TGT CTT GTG ACT GGA −3′; *Trpv1* Fw, 5′- ACT CAC CAT CGC CGC CTA CTA C −3′; *Trpv1* Rv, 5′- ACG CTT CAG TTG GCA GCA TCG G −3′; *Trpv3* Fw, 5′- GCM TGG TTC CAC TTY GCA TT −3′; *Trpv3* Rv, 5′- CTG TTG GCC CTG GRT CTT CAT −3′; *Trpv4* Fw, 5′- AGG GAA GAC STG CCT GCC CAA −3′; *Trpv4* Rv, 5′- CGT AYG CCC ART CCT TGA ACT T −3′.

### Electrophysiological recordings

All five Nile crocodiles (*C. niloticus*) and five spectacled caimans (*Caiman crocodilus*) used in this experiment were juvenile individuals (700 g to 1,200 g). Briefly, animals were anesthetized by intramuscular injection of alfaxolone (10 mg/kg; Alfaxan). An adhesive ground electrode was placed under the belly to reduce electrical interference, and four needle electrodes (one reference and three recording electrodes) were implanted subcutaneously (via the soft inter-scale region of scales) into the dermal component of distinct sensory organs along the jaw (for crocodiles and caimans) or dorsal skin (for crocodiles). The correct implantation of electrodes was controlled by induction of electrical activity of the ISO upon gentle brushing stimulation. Different local stimuli were applied to the skin region bearing the recording electrodes, and the voltage difference between the reference and each recording electrode (common reference method) was recorded using a NicoletOne multichannel, high impedance amplifier/electroencephalogram system (Neuroswiss, Bern. Switzerland). The following stimuli were used: mechanical (manual brushing of non-ISO- and ISO-bearing skin regions under dry and wet conditions), thermal (local applications of heat (25°C to 55°C) and cold (10°C to 25°C) using a heat lamp and an ice pack, respectively), pH (solutions of 0.01 M HCl and 0.01 M NaOH applied as droplets directly on ISOs), chemical (1 mM 2-APB applied as droplets directly on ISOs), and hyperosmotic salinity (solutions of 0.5 M NaCl and 0.5 M KCl applied as droplets directly on ISOs). The reference skin region was either non-stimulated (control of mechanical and thermal stimuli under dry conditions) or exposed to water (control of mechanical, pH and hyperosmotic stimuli under wet conditions). The electrical activity of individual sensory ISO organs was subsequently visualized and analyzed using EEGVue and NicVue software (Nicolet Biomedical, Madison, WI, USA).

## Results

### Distribution and structure of cutaneous sensory micro-organs in crocodylians

**Figure 1:**
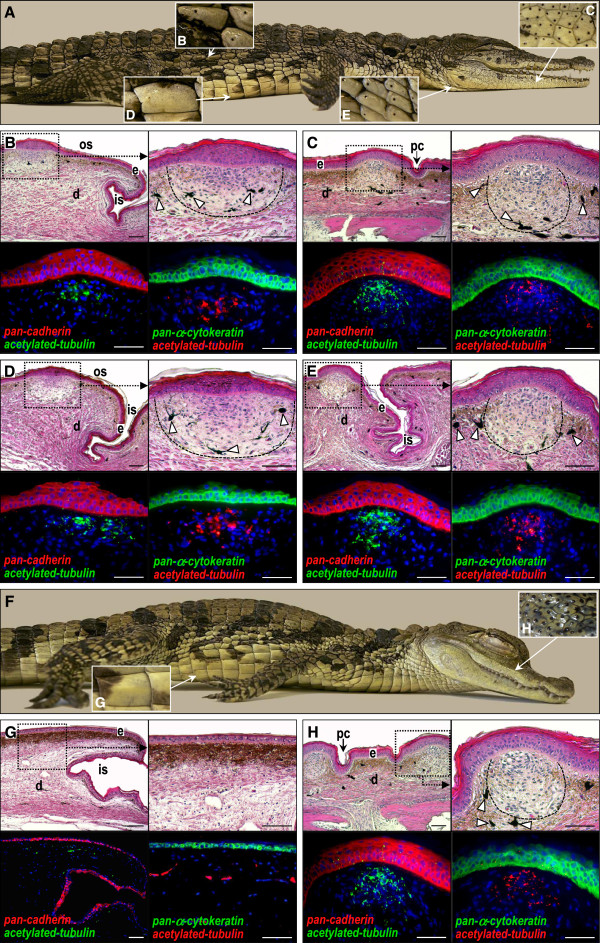
**Distribution and structure of integumentary sensory organs in crocodylians. (A)** Anesthetized juvenile *C. niloticus* showing the presence of integumentary sensory organs (ISOs) on dorsal, face/jaws, ventral, and neck scales (insets **B-E**). **(B-E)** Parasagittal sections of corresponding ISO-bearing skin stained with H&E (low and high magnifications) or processed for double immunostaining of acetylated tubulin (neuronal marker) together with pan-cadherin (general epithelial marker) or pan-α-cytokeratin (α-keratin-specific epithelial marker). Cell nuclei were counterstained with 4',6-diamidino-2-phenylindole (blue). **(F)** Anesthetized juvenile *Caiman crocodilus* showing the absence of ISOs on postcranial scales (inset **G**) and their presence on face/jaws scales only (inset **H**). **(G-H)** Parasagittal sections of corresponding skin processed as in **(B-E)**. The lightly stained dermal component of ISOs is indicated by dashed lines. White arrowheads indicate melanocytes; e, epidermis; d, dermis; os, outer-scale region; is, inter-scale region; pc, primary crack. Magnification bars: 100 μm.

To better describe both the general distribution and structure (in terms of epidermal stratification and dermal component) of ISOs throughout the body of crocodylians, we first used standard histology and immunohistochemistry methods to analyze the skin from different body regions (dorsum, ventrum, neck, jaws, oral mucosa) in juvenile Nile crocodile (*C. niloticus*) and spectacled caiman (*Caiman crocodilus*) individuals. Gross morphological examination of the skin first showed that the shape, thickness, size, degree of overlap, and pigmentation pattern of scales vary in different body regions, but these variations are relatively well-conserved between crocodiles and caimans (Figure 1A, F). The skin is hardest in dorsal and nuchal scales, which contain osteoderms and exhibit conspicuous ridges. The lateral, ventral, and neck skin is more flexible and consists of large, flat, roughly square-shaped scales (orderly arranged in rows on the ventral part). ISOs are visible as small, melanin-pigmented spots restricted to the head in caimans, but distributed all over the body (including on postcranial scales in the dorsal, ventral, leg, and cloacal regions) in Nile crocodiles (Figure 1A, F). While only one ISO is present per postcranial scale, the number of these organs on cranial scales is highly variable (ranging from one to about thirty). These differences are due to selection (denser distribution of sensory organs on the head is adaptive) and fundamentally different developmental mechanisms between facial/jaw scales and postcranial scales [11]. The relative density of ISOs is yet higher in the proximity of the teeth and lower at the back of the jaws, as well as on the top of the head and face. The absolute number of ISOs can differ by as much as 21% among Nile crocodiles [11] and is not correlated to the size of the animals (that is, smaller animals tend to exhibit a more compact array of ISOs).

Histological comparison of Nile crocodile skin from different body regions revealed a variably thick stratified epidermis, ranging from 40 μm to more than 100 μm on postcranial and jaw scales, respectively (Figure 1B, C, top panels). We also observed additional α-keratin layers in the epidermis immediately overlying the ISO in all dorsal, ventral, and neck scales, as shown by immunostaining of both general pan-cadherin and α-keratin-specific epithelial markers (Figure 1B, D, E, lower panels). Also, the outer layer, the *stratum corneum,* is thinner and more compact in the ISO region, when compared to the rest of the integument, suggesting that the epidermal barrier function is modified in the former region (Figure 1B, D, E). On Nile crocodile jaws, the epidermis in the non-ISO region is about 2-fold thicker than on the rest of the body (Figure 1C), but both the number of suprabasal layers and the thickness of the *stratum corneum* are specifically decreased in the ISO region. In other words, while the thickness (and organization) of the epidermis in the non-ISO regions is very different between postcranial and cranial scales, the ISO region is very similar in all scales. Within the dermis, the ISO includes an ellipsoidal (in dorsal and ventral regions) or spherical (in jaw and neck regions) space immediately underlying the modified epidermis (Figure 1B-E, top panels). This dermal pocket shows very few collagen fibers but contains several cell types, including fibroblasts, melanocytes, and highly branched nerve terminals (the latter are identified by immunostaining with the general neuronal marker acetylated tubulin; Figure 1B-E). In spectacled caimans, we observed epidermal and dermal compartments of cranial ISOs similar to those in Nile crocodiles (that is, a reduced number of epidermal layers and spherical dermal pocket of multiple cell types; Figure 1H). However, postcranial scales, in the absence of ISOs, showed a more constant epidermis, as well as nerve terminals widely dispersed among dermal collagen fibers (Figure 1G).

**Figure 2:**
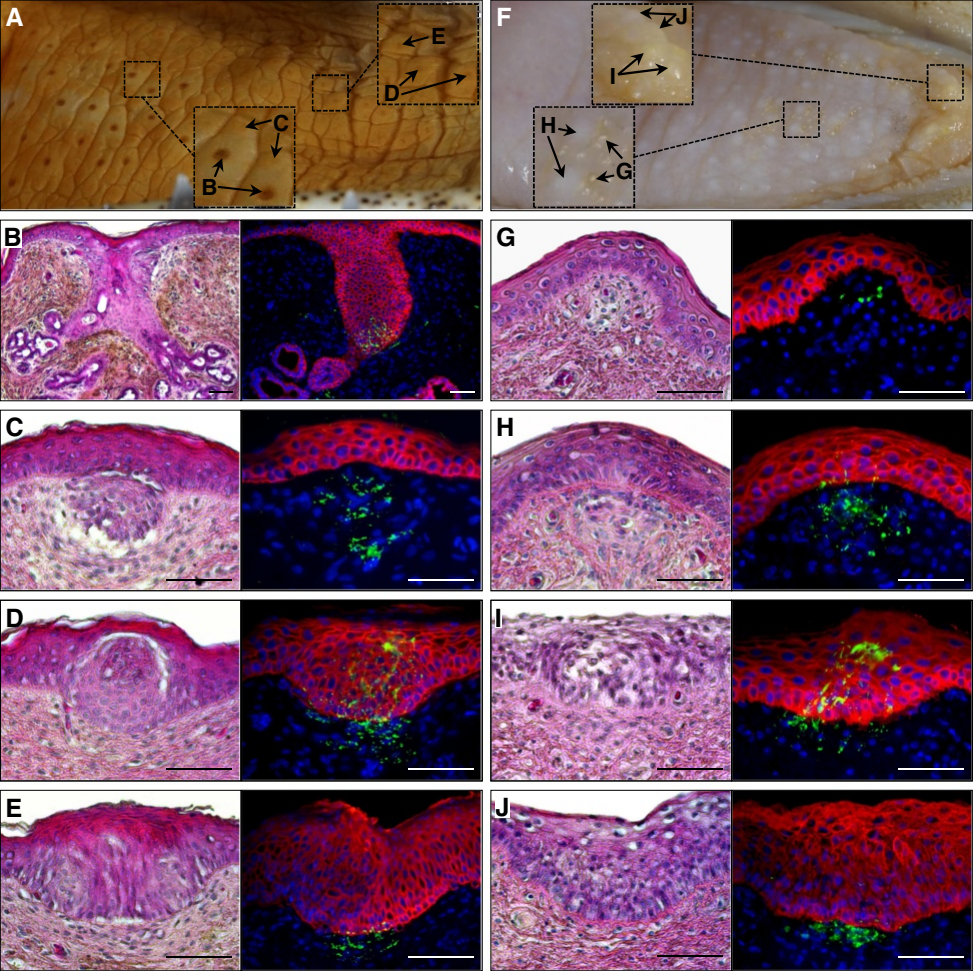
**Distribution and structure of lingual sensory organs in crocodylians.** Macrophotographs of the *C. niloticus***(A)** and *Caiman crocodilus***(F)** tongue showing the presence of different types of organs over its surface: salt glands **(B)**, large **(C and H)** and small **(G)** ISO-related structures, taste buds **(D and I)**, and large epithelial thickenings **(E and J)**. Parasagittal sections were stained with H&E (left panels) or processed for double immunostaining (right panels) of pan-cadherin (general epithelial marker, red) and acetylated tubulin (neuronal marker, green). Cell nuclei were counterstained with 4',6-diamidino-2-phenylindole (blue). Magnification bars: 100 μm.

Stereomicroscopic and histological examination of the oral cavity in the two crocodylian species also revealed marked differences in both the epithelial organization and the collection of lingual structures. As already reported in different crocodylids [31,32,35,41], we observed that the tongue of Nile crocodiles exhibits a relatively smooth surface punctated by 40 to 50 large, darkly pigmented salt glands situated on its posterior two-thirds (Figure 2A). When sectioned, these glands appear as compound, branched, tubular structures composed of a thick, epithelial secretory duct containing acetylated tubulin-positive neurons (Figure 2B). Whereas the lingual epithelium of the spectacled caiman lacks salt glands (Figure 2F), we show that it exhibits multiple non-pigmented dome-like structures of two size classes (small and large), randomly distributed on the whole tongue surface, and extremely similar to the cranial ISOs in terms of morphology and neuronal distribution (Figure 2F-H). Interestingly, we found only large ISO-related units on the Nile crocodile tongue, including in between the salt glands (Figure 2C). Finally, in both crocodiles and caimans we identified other types of less conspicuous surface units on the lateral and apical regions of the tongue, including ellipsoid intraepithelial structures resembling taste buds [33,35], as well as large epithelial thickenings with a compact cluster of neurons restricted to the underlying connective tissue (Figure 2D, E, I, J).

Altogether, these results indicate that both the integumentary and oral sensory organs are relatively well-conserved between crocodylids and caimans. The major differences include the absence of both postcranial ISOs and lingual salt glands in caimans, suggesting a reduced capacity of these animals to sense specific variations in the external environment.

### Embryonic development of cranial, postcranial, and lingual sensory units

**Figure 3:**
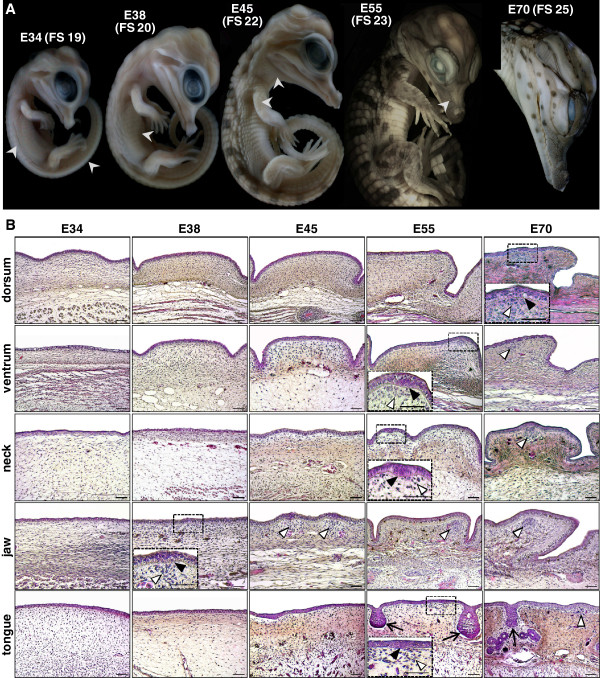
**Development of cutaneous sensory structures during crocodylian embryogenesis. (A)** Lateral views of developing *C. niloticus* embryos. Embryonic days **(E)** post-oviposition, and corresponding Ferguson stages (FS) of development, are indicated on top. White arrowheads indicate the initiation of scale formation on the different body regions. **(B)** H&E staining of skin sections from different body regions (indicated on the left) of *C. niloticus* embryos at various developmental stages (E34 to E70). Black frames indicate the first appearance of ISOs (insets: higher magnifications). White and black arrowheads indicate the dermal and epidermal components of developing ISOs, respectively. Arrows in bottom panels mark developing lingual salt glands. Magnification bars: 100 μm.

Below, we describe the morphogenesis time course of the skin and associated sensory organs in Nile crocodile embryos. Figure 3A shows that scale development is not synchronous across the body of Nile crocodile embryos, as already observed in alligators [4]. Scale morphogenesis initiates on the tail and dorsum at embryonic day E34 (that is, 34 days post-oviposition, corresponding to FS 19 [36,37]), on the ventrum at E38 (FS 20), and then on the neck and limbs at E45 (FS 22), through a conserved mechanism involving undulations of the entire skin surface (that is*,* the epidermis and dermis), followed by the production of symmetrical dermo-epidermal elevations that become progressively asymmetrical depending on their final degree of overlap (Figure 3B). In contrast, crocodylian face- and jaw-scales never form from such developmental stages [11]: instead, these scales are generated (starting from E55 = FS 23) through the formation of grooves that propagate and interconnect (Figure 3A and [11]). We show here that ISOs on the face and jaws are the first cutaneous sensory organs to develop during Nile crocodile embryogenesis (from E38; Figure 3B). Consequently, the pattern of ISOs on the head is fully formed when head scales start to form (around E55) by cracking of the developing skin. We observed the same relative timing of development in caiman embryos (data not shown). In contrast, the formation of postcranial ISOs is initiated at different embryonic stages across the body and at different stages of scale morphogenesis. For instance, the formation of ISOs was clearly visible in the neck scales when they were still symmetrical (at E55), while it was observed when scales started to become asymmetrical in the ventral region (E55) or when they had basically completed their asymmetrical development (at E70 or FS25) in dorsal scales (Figure 3B). Finally, we show here that the developmental process leading to ISO formation is remarkably similar in all body regions (including the jaws and tongue): a local thickening of the epidermis first appears (Figure 3B; black arrowheads) together with condensation of the underlying dermal cells (white arrowheads). The molecular mechanisms underlying ISO morphogenesis remain to be identified, but our data suggest the critical involvement of dermo-epidermal reciprocal interactions in this process, similarly to the initial morphogenesis of epithelial appendages in vertebrates.

Together, these results show that the formation of ISOs across the body of Nile crocodiles is not synchronized with the process of scale morphogenesis, in agreement with the existence of very different mechanisms responsible for generating postcranial and cranial scales in crocodylians.

### Innervation pattern of ISOs is conserved in crocodylians

**Figure 4:**
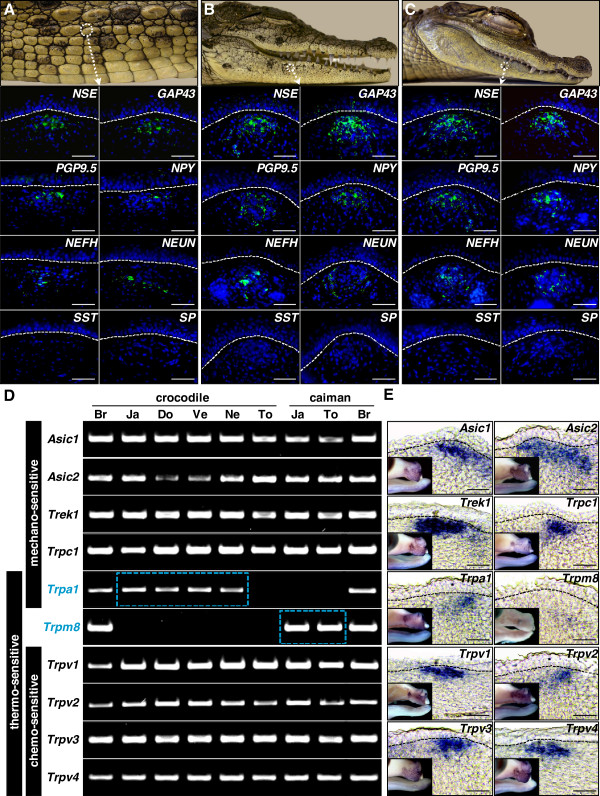
**Innervation patterns of integumentary sensory organs in crocodylians.** Macro-photographs of the Nile crocodile trunk **(A)** and head **(B)**, and spectacled Caiman head **(C)**. Lower panels show parasagittal sections of the corresponding integumentary sensory organs (ISOs) processed for immunostaining of various neuronal markers: neuron-specific enolase (NSE), growth-associated protein 43 (GAP43), neuron cytoplasmic protein gene product 9.5 (PGP9.5), neuropeptide Y (NPY), neurofilament, heavy polypeptide (NEFH), neuron-specific nuclear protein (NEUN), somatostatin (SST), or substance P (SP). Cell nuclei were counterstained with 4',6-diamidino-2-phenylindole (blue). The epidermal-dermal junction is indicated by dashed lines. Magnification bars, 100 μm. **(D)** Semiquantitative reverse transcription-PCR analysis of genes (names indicated on the left), encoding transduction channels with different sensory modalities (vertical black rectangles), in Nile crocodile and spectacled caiman ISO-bearing skin tissues from different body parts (Ja, jaws; Do, dorsum; Ve, ventrum; Ne, neck; To, tongue). The embryonic brain (Br) of crocodylian species was used as a positive control for gene expression. Blue dashed rectangles indicate differential expression of *Trpa1* and *Trpm8* in Nile crocodile and spectacled caiman. **(E)** Parasagittal cryosections of ISOs stained using whole-mount *in situ* hybridization with probes targeting various ion channel genes (indicated on the corresponding panel) on embryonic Nile crocodile heads at E45 (small insets). The epidermal-dermal junction is indicated by dashed lines. Magnification bars: 100 μm.

Cutaneous sensory neurons activated by mechanical, temperature, and damage (pain) stimuli, are remarkably diverse in their degree of myelination, conduction velocity, and sensory modality [42]. Also, different types of neural proteins are expressed by specific neurons, but relatively little is known about the neuronal innervation of sensory organs in reptiles, except for a few reports on the salt glands of turtles and crocodylids [43-45]. To compare the innervation pattern of sensory organs in Nile crocodiles and spectacled caimans, we first analyzed by immunohistochemistry, the density and distribution of several major neural proteins (namely*,* neuron-specific enolase (NSE); growth-associated protein 43 (GAP43); neuron cytoplasmic protein gene product 9.5 (PGP9.5); neurofilament, heavy polypeptide (NEFH); and neuron-specific nuclear protein (NEUN)), and neuropeptides (namely, neuropeptide Y (NPY); somatostatin (SST); and substance P (SP)) within the nerve terminals of ISOs. Figure 4A-C indicates that neurons containing NSE, GAP43, PGP9.5, NPY, NEFH and NEUN are abundant in all three types of ISO (crocodile postcranial and cranial, caiman cranial), with similar higher density in the dermal pocket and lower density in the epidermal component. These proteins are also widely expressed in the peripheral nervous system of other amniotes, including mammals [46,47]. Finally, although immunostaining identified the presence of SST and SP proteins in the embryonic brain of the *Crocodylus* species (data not shown), these two neuropeptides were not detected in crocodylian ISOs (Figure 4A-C).

We next explored the nature and distribution of sensory receptors in the Nile crocodile and spectacled caiman ISOs. Sensory receptors are defined here as sensory neuron endings that respond to a specific stimulus in the internal or external environment. Various types of mechano-, thermo- and chemoreceptors are specialized to provide information to the central nervous system about mechanical, temperature, and chemical/pH variations, respectively. These stimuli are detected by specific transduction channels that are expressed in the plasma membrane of sensory receptors in vertebrates [42]. Thus, we examined, both with semiquantitative RT-PCR and WMISH, the expression patterns of different conserved families of transduction channels (that is, acid-sensing ion channels (*ASIC*); potassium channel subfamily K (*TREK*); and transient receptor potential (*TRP*)) in ISO-bearing crocodylian skin tissues. Our RT-PCR on ISO-bearing skin from jaws, dorsum, ventrum, neck, and tongue indicate a similar positive expression of all tested mechanoreceptor (*Asic1*, *Asic2, Trek1* and *Trpc1*), as well as polymodal chemo- and thermoreceptor channels (*Trpv1*, *Trpv2*, *Trpv3* and *Trpv4*) in crocodiles and caimans (Figure 4D). Conversely, we found significant species-specific differences in the expression pattern of both *Trpa1* and *Trpm8* genes, the two only known transduction channels responsive to cold temperature in mammals [42]. While *Trpa1* was detected in postcranial and cranial scales (but not in the tongue) of Nile crocodiles and not detected in caimans, *Trpm8* is specifically expressed in the jaw-skin and tongue of caimans, suggesting that different crocodylian species use different peripheral thermoreceptor channels (Figure 4D). Finally, using WMISH with species-specific antisense riboprobes (sense strand used as negative controls), we show in Figure 4E that all transduction channels identified by RT-PCR are specifically expressed in developing ISOs during embryogenesis and nowhere else in the skin. This discretization of skin sensory expression islands (ISO-specific) is unique among vertebrates. For example, it strongly differs from the situation in mammals, where sensory neurons and transduction channels are diffusely distributed across the skin [48].

### Multi-sensorial functions of crocodylian ISOs

**Figure 5:**
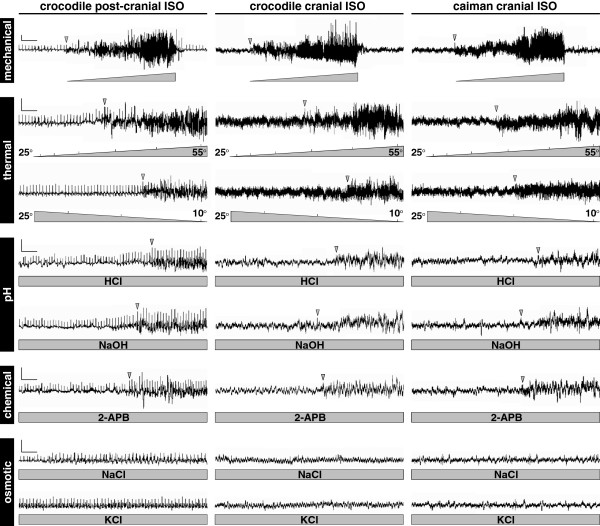
**Electrophysiological recordings of crocodylian integumentary sensory organs.** Representative traces of electrical activity of Nile crocodile postcranial and cranial integumentary sensory organs (ISOs) (left and middle panels), and spectacled caiman cranial ISOs (right panels) in response to: increasing mechanical stimulus; increasing (from 25°C to 55°C) or decreasing (from 25°C to 10°C) thermal stimuli, acidic (HCl) or alkaline (NaOH) pH stimuli; chemical stimulus (2-APB); and hyper-osmotic stimuli (NaCl or KCl). Gray triangles (for increasing or decreasing stimuli) and rectangles (for constant stimuli) indicate the intensity and period of stimulation. Gray arrowheads indicate the beginning of visible positive responses. Electrophysiological measurements were performed on single ISOs. Scale bars: vertical, 10 μV; horizontal, 10 s.

The simultaneous presence of various types of mechano-, chemo- and thermosensory transduction channels in crocodylian ISOs strongly suggests that they might be involved in multiple functions, in addition to previously identified surface wave detection [26,27]. To test our hypothesis, we performed *in vivo* extracellular recordings from individual ISOs in juvenile Nile crocodiles and spectacled caimans, to assess the sensory modalities of postcranial and cranial ISOs using different local stimuli that naturally occur in their environment (that is, changes of pressure, temperature, salinity and pH). First, we confirmed previous observations reported in alligators and crocodylids [26,27]: cranial ISOs in caimans and crocodylids, but also postcranial ISOs in crocodylids, are able to detect mechanical stimuli, here induced by brushing (Figure 5). The amplitude of the electrical responses was positively correlated with the intensity of the stimulus. However, we further demonstrate that all types of ISOs additionally respond to both warm and cold temperatures. Note that ISOs are sensitive beyond a certain temperature threshold, as their response to a gradual temperature increase or decrease was not visible until the temperature reached approximately >43°C and <15°C, respectively (Figure 5). The response to temperature stimuli then increased proportionally with higher and lower temperatures from 43°C to 55°C and from 15°C to 10°C, respectively. Note also that Nile crocodile ISOs systematically demonstrated a colder threshold to cooling than their caiman counterparts, possibly deriving from the presence of different types of thermosensitive channels in these two species (as demonstrated above; Figure 4D). Importantly, these response profiles are coherent with published data on the activation of thermosensory transduction channels of the *TRP* family at various temperature thresholds in mammalian cells, ranging from below 18°C to 52°C [42]. We interpret that the successive activation of different types of channel explains the amplification of the electrophysiological response of ISOs during a gradual increase in temperature. In addition, we suspect that the low number of transduction channels activated before a certain temperature threshold results in low or undetectable positive electrical responses from individual ISOs.

To further test the multi-sensory abilities of ISOs, we next examined their responses to other stimuli, including acidic pH (HCl treatment), alkaline pH (NaOH), *TRPV* chemical agonist (2-aminoethoxydiphenyl-borate (2-APB)), and hyper-osmotic salinity (NaCl and KCl). Although the electrophysiological responses were globally lower than for mechanical and thermal stimuli, we show that ISOs are all clearly sensitive to both increase and decrease of pH after a latency period (Figure 5). The low diversity of peripheral sensory transduction channels responsive to variations of pH, when compared to other stimuli, could explain the low response of crocodylian ISOs. Indeed, only a few members of the *TRP* family, including *TRPV1* and *TRPV3*, have been shown to respond to pH changes in mammalian cells [49,50]. We additionally observed that the stimulation of ISOs with the specific *TRPV1-3* agonist 2-APB triggered a similar response time to acidification or alkalinization (Figure 5), suggesting that these transduction channels might also act as pH sensors in crocodylians. The observed latency period upon acidic and alkaline pH stimulations most likely derived from the progressive and/or slow activation of *TRPV* channels. Finally, we did not detect any electrophysiological response of Nile crocodile and caiman ISOs to hypertonic NaCl or KCl concentrations (equivalent to seawater), even after a prolonged period (Figure 5). This suggests that the abilities of some crocodylians to discriminate salinity [28,51] is not associated to ISOs but to other sensory organs that remain to be identified.

Altogether, these results demonstrate that postcranial and cranial ISOs of crocodylians combine a wide array of mechanical, thermal and pH sensitivities, making them remarkable multi-sensorial micro-organs with no equivalent in the sensory systems of other vertebrate lineages.

## Discussion

Here, we report on the development and function of integumentary organs in crocodylians. These micro-organs all exhibit similar morphology and mode of development during embryogenesis, although they differ in distribution, density, and timing of formation among scales in different regions of the body, especially in crocodylids (represented here by the Nile crocodile). Most importantly, our molecular analyses indicate that sensory neurons of crocodylian ISOs express a large repertoire of transduction channels involved in mechano-, thermo-, and chemosensory functions. We confirm these results with electrophysiological analyses showing that each ISO exhibits a combined sensitivity to mechanical, thermal and pH stimuli. Altogether, these data indicate that the common ancestor of all crocodylians transformed the ancestral diffused sensory system of the skin (still present in all other vertebrates) into an array of discrete, multi-sensory micro-organs innervated by multiple pools of cutaneous sensory neurons. In addition, our analyses (and others: [27]) indicate that ISOs are unable to respond to osmotic stimuli, suggesting that the ability of crocodylids to discriminate salinity is most likely due to the presence of sensory organs (possibly the salt glands themselves) in their oral cavity.

### Crocodylian ISOs are globally adapted to natural stimuli

Extant members of the crocodylian order are represented by at least 23 species, occupying a range of semi-aquatic environments in both temperate and tropical regions of North and South America, Africa, Asia, and Australia [52]. Whereas most crocodylians are found in fresh water, all crocodylids, but not alligators and caimans, can adapt to hyperosmotic conditions, and crocodylid populations and species occur routinely in estuarine habitats. Adaptations to salty water include reduced integument permeability [53], modified buccal epithelium [54], lingual salt-secreting glands [32], and behavioral osmoregulation [28,51]. It is unknown if the salt glands observed in gharials (genera *Gavialis* and *Tomistoma*), which exclusively inhabit fresh water, would allow them to adapt to salty water, or if they constitute a relict trait hinting at their sister relationship with crocodylids. We show here that postcranial ISOs in Nile crocodiles, not present in alligators and caimans but present in gharials,, do not provide function associated with life in saline environments.

Behavioral experiments have shown that cranial ISOs are able to detect surface pressure waves, allowing crocodylians to swiftly orient towards disturbances of the water-air interface [26,27], hence contributing to prey localization when underwater vision is poor [55]. Although we confirm here that Nile crocodiles and spectacled caimans possess structurally and functionally similar ISOs responsive to mechanical stimuli, we further demonstrate that these structures also respond to temperature and pH stimuli, consistent with the local modifications of the epidermal barrier permeability (that is, thinner *stratum corneum*) in these regions. These additional functions of ISOs are likely advantageous to face environmental variations that occur daily and seasonally in both temperate and tropical environments. For example, given their ectothermy, crocodylians display typical patterns of physiological and behavioral thermoregulation, the latter consisting of periodic movement between heating and cooling sources [56-58]. Differences in both thermal behavior and tolerance to extreme temperatures between crocodylid and alligator/caiman species have been observed [59], suggesting that the different distribution pattern of ISOs may have an impact on their thermoregulatory response. Similarly, in addition to lingual salt glands that allow adaptation to hypertonic environments, the presence of postcranial chemosensitive ISOs in *Crocodilydae* is likely to confer them an advantage over alligators and caimans in adapting to environments that differ in pH, temperature, and chemical composition. Thereby, we suspect that these sensory organs may have contributed (along with other natural and anthropogenic factors) to the wider geographical distribution of extant crocodylids, in contrast to the more specific habitat of alligators and caimans.

### ISOs are richly innervated by different types of conserved sensory neurons

All vertebrates possess different types of skin sensory neurons that transduce mechanical, thermal, and pH stimuli into action potentials that propagate to the central nervous system. Different sensory modalities are governed by the specific expression of separate transduction channels in these neurons [42]. Our study demonstrates that the observed functional specialization (namely, mechano-, chemo-, and/or thermosensitive) and level of sensitivity (low activation threshold for mechanoreception and variable for others) of these channels in mammals [42] are conserved in crocodylians. For example, members of the *TRP* family of cation channels are polymodal signal detectors that respond to a wide variety of physical and chemical stimuli, making them important components of sensory systems in both vertebrates and invertebrates. Of the six mammalian *TRPV* channels, *TRPV1* to *TRPV4* were demonstrated to be involved in the transduction of thermal stimuli [42]. In cell culture models, *TRPV1* and the related receptor *TRPV2,* are activated by high temperatures (above 42°C and 52°C, respectively), whereas *TRPV3* and *TRPV4* are activated by lower temperatures (27°C to 34°C and 34°C to 39°C, respectively). In addition to warm temperature, *TRPV1* and *TRPV3* have been shown to respond to severe pH changes, while *TRPV2* and *TRPV4* are activated by cell swelling induced by hypotonic solutions [49,50,60]. Our study suggests that similar *TRPV* channels are used by crocodylian ISOs to detect different temperature and pH thresholds. Other *TRP* channels known to contribute to thermosensing in mammals, but also in reptiles [61], include *TRPM8* and *TRPA1*, two important molecular mediators of thermoperception with a different threshold. Note that the differential expression of these channels in ISOs of crocodylids and caimans is consistent with the observed differences in both thermoregulatory behavior [59,61] and cold sensitivity (our study) between these species. In addition, the presence in Nile crocodile ISOs of *TRPA1* (rather than *TRPM8*), a channel that exhibits a unique dual function (thermo- and mechano-sensorial) might contribute (together with the presence of postcranial ISOs) to increase the overall sensitivity of the skin in crocodylids versus caimans and alligators. Finally, we show that all other mechanoreceptor channels previously identified in low-threshold cutaneous neurons of mammals, including *TRPC1*, *ASICs* and *TREK-1*[62,63], are also present in crocodylian ISOs.

Although the different types of sensory transductions in the skin seem conserved between mammals and crocodylians, the distribution pattern of cutaneous sensory neurons is not. In mammals, mechano-sensitive and thermo-sensitive neurons are diffusely distributed across the skin (in the epidermis, dermis, and/or subcutaneous tissue), whereas chemo-sensory neurons are found exclusively on the tongue as part of sensory organs called taste buds. In contrast, in ISO-bearing skin regions of crocodylians, these different populations of neurons are all grouped into the ISOs and are absent from the skin in between the ISOs. On the other hand, our results indicate that sensory neurons are diffusely distributed in the postcranial skin of spectacled caimans, that is, in the region of the body lacking ISOs.

### ISOs are unique conserved sensory systems of semi-aquatic crocodylians

Although vertebrates have evolved multiple specialized cutaneous sensory structures that vary in morphology, distribution, and function among taxa, crocodylian ISOs appear to be the most complex arrangement of different types of sensory neurons, despite their external resemblance (dome-like structures) with more specialized mechano-sensory organs such as the push-rod cutaneous organs of monotremes [64], the Eimer’s epidermal organ of star-nosed moles [65], and the bill tip organ of birds [66]. Other reptilian species, including lizards, snakes, and tortoises, also possess integumentary sensory structures over most of their body, but only a few studies have formally investigated their function(s). These sensory organs are, therefore, usually described by default as mechanoreceptors, even in the absence of any physiological evidence. The morphology of these structures in *Iguanidae* and *Agamidae* lizards has been shown to involve both dermal and intra-epidermal modifications [14,17,67]. Similarly, different types of cutaneous sensory receptors have been described within scales bordering the toes of gecko lizards [68]. Less attention has been given to the integumentary sensory system of tortoises, except for the description of dermal sensory corpuscles in the facial skin of the Russian tortoise [19]. In addition, the identification of only a few thermo-sensitive nerve fibers in the reptilian skin [69] might be explained by the presence of more specialized highly sensitive thermoreceptor organs, including the pineal gland of the brain in all non-archosaurian reptiles [70], as well as the infrared-sensitive pit organs located on each side of the face between the nostril and the eye in vipers [21], and on labial scales in some pythons and boas [16,71].

Our analyses of ISOs in crocodylians constitute the first report of an integumentary chemo-sensory organ in an amniote vertebrate. Indeed, except for desert toads that were shown to detect hyperosmotic salt solutions with the ventral skin [72], chemoreceptors in vertebrates have been described exclusively as taste buds in the oral cavity. ISOs, however, supplement rather than replace taste buds, as the latter are also present in the lingual mucosa, palate, and gingivae of crocodylian species as shown previously [34], and in this study. Note that compound sensory structures, each probably comprising taste buds and various mechano-sensory corpuscles, have also been identified along the tooth rows in some species of land snakes [18]. Although the ability of this oral sensory organ to receive both chemical and mechanical information has not been functionally investigated, it suggests that in addition to crocodylian ISOs, other multifunctional sensory systems could exist in other reptilian lineages.

 Finally, many marine non-mammalian amniotes possess specialized salt-secreting glands capable of maintaining osmotic homeostasis in hyper-osmotic environments. These extrarenal salt glands have evolved independently in various lineages, and are lingual in crocodylids, sublingual in sea snakes, lachrymal in sea turtles, nasal in marine iguana, and supraorbital in sea birds, but they are all richly innervated by neurons and are comparable in ultrastructure as shown previously [73], and in this study. Changes in plasma osmotic concentration can be detected by plasma volume baroreceptors and cerebral osmoreceptors [73], triggering salt gland activation. However, our study suggests that in addition to the behavioral osmoregulation reported in crocodylids (such as, avoiding drinking sea water [28]), salt glands (but not ISOs) could be direct salinity sensors. Further experiments on the tongue mucosa, including electrophysiological recordings of potential salt-sensitive neurons, as well as investigation of more specialized salt-sensing pathways (for example, the ENaC epithelial sodium channel), will be necessary to confirm this hypothesis.

## Conclusions

This study shows that crocodylian ISOs all exhibit similar morphologies and modes of development during embryogenesis, despite differences in their distribution, density, and timing of formation among scales across the body. Most importantly, we suggest that ISOs exhibit a combined sensitivity to mechanical stimuli, cold and warm temperatures, and pH variations, making them remarkable multi-sensorial micro-organs with no equivalent in the described sensory systems of other vertebrate lineages. Finally, our analyses indicate that ISOs are unable to respond to osmotic stimuli, suggesting that the ability of some crocodylians to discriminate salinity is associated to other sensory organs in their oral cavity. Altogether, this study indicates that the ancestral vertebrate diffused sensory system of the skin was transformed in the crocodylian lineage into an array of scattered, discrete, multi-sensory micro-organs innerved by multiple pools of sensory neurons. This discretization of skin sensory expression islands is unique among vertebrates, and allowed crocodylians to develop highly armored, but very sensitive skin.

## Abbreviations

2-APB: 2-aminoethoxydiphenyl-borate; ASIC: Acid-sensing ion channel; Bp: Base pairs; DAPI: 4',6-diamidino-2-phenylindole; DPR: Dome pressure receptor; FS: Ferguson stage; GAP43: Growth-associated protein 43; H&E: Hematoxylin and eosin; ISO: Integumentary sensory organ; NEFH: Neurofilament heavy polypeptide; NEUN: Neuron-specific nuclear protein; NSE: Neuron-specific enolase; NPY: Neuropeptide Y; OCT: Optimum cutting temperature; PBS: Phosphate-buffered saline; PFA: Paraformaldehyde; PGP9.5: Neuron cytoplasmic protein gene product 9.5; RT-PCR: Reverse transcription polymerase chain reaction; SP: Substance P; SST: Somatostatin; TREK: Potassium channel subfamily K; TRP: Transient receptor potential; WMISH: Whole-mount *in-situ* hybridization

## Competing interests

The authors declare that they have no competing interests.

## Authors’ contributions

ND and MCM conceived and designed the experiments. ND performed the experiments. ND and MCM analyzed the data and wrote the paper. Both authors read and approved the final manuscript.
